# ‘Continuity of care’: a critical interpretive synthesis of how the concept was elaborated by a national research programme

**DOI:** 10.5334/ijic.794

**Published:** 2012-04-13

**Authors:** Janet Heaton, Anne Corden, Gillian Parker

**Affiliations:** Previously Social Policy Research Unit, University of York, York, North Yorkshire, YO10 5DD, UK and Currently Institute of Health Services Research, Peninsula College of Medicine and Dentistry, Universities of Exeter and Plymouth, The Veysey Building, Salmon Pool Lane, Exeter, Devon, EX2 4SG, UK; Social Policy Research Unit, University of York, York, North Yorkshire, YO10 5DD, UK; Social Policy Research Unit, University of York, York, North Yorkshire, YO10 5DD, UK

**Keywords:** continuity of care, partnerships, critical interpretive synthesis, meta-ethnography, qualitative synthesis

## Abstract

**Introduction:**

A Continuity of Care Research Programme was undertaken in England in 2000–9. The Programme was informed by a conceptual framework proposed by Freeman and colleagues in an earlier scoping study. At the end of the Programme, a conceptual synthesis was carried out in order to confirm or refine the ‘Freeman model’ of continuity of care.

**Methods:**

A conceptual synthesis of the outputs of the Programme, using Critical Interpretive Synthesis.

**Results:**

The conceptual framework underpinning the Freeman model of continuity of care, which prioritises the perspectives of service users and carers, was variously utilised in the Programme. Analysis revealed indications of an emerging shift from the patient and carer ‘perspectivist’ paradigm of the Freeman model towards a new ‘partnership’ paradigm where continuity is recognised to be co-constructed by patients, families and professionals, all of whom have an active part to play in its accomplishment.

**Conclusions:**

The projects in the Programme have advanced understanding of patients’ perspectives on continuity of care and on the complex nature of this concept. At the same time, they have raised issues and reported findings which may be indicative of an emergent paradigm shift in this area of research, towards a more dynamic partnership model.

## Introduction

Despite continued attempts to alter policy and change practice, the ability of health and social care systems to deliver the type and level of continuity of care that service users desire remains in question. Lack of clarity about what continuity of care actually means, as well as imperfections in systems to deliver it, have been identified as part of the cause of this problem. In 2000–9, the English National Institute for Health Research Service Delivery and Organisation (NIHR SDO) funded a series of research projects, both primary and secondary, on continuity of care, specifically to tackle this conceptual confusion [1]. This Continuity of Care Programme also aimed to add to the knowledge base about what service users want in the way of continuity of care, what influences whether or not they experience it and the outcomes it may produce.

The Programme began with a scoping study, carried out in 2000–1 by George Freeman and colleagues, reviewing the literature on continuity of care and analysing the concept [2]. In the report of this work, a new conceptual framework for understanding continuity of care was advanced and this formed the basis for the Programme. In the ‘Freeman model’, as it became known, patients’ and carers’ experiences of continuity of care (so-called ‘experienced continuity’) are absolutely key; from their perspective, whether ‘experienced continuity’ is positively achieved or not depends on how well services perform on particular dimensions that contribute to this experience.

Following the scoping study, six large primary studies and three reviews were commissioned and successfully completed between 2001 and 2008 (see Table 1). These projects used mixed methods to examine patients’ and carers’ experiences of continuity of care in relation to different condition groups and/or services, and to develop and test measures of ‘experienced continuity’. Freeman and colleagues were also commissioned to conduct a synthesis of the Programme’s outputs. However, when they carried out this work in 2007, only three of the six primary studies had completed, hence they mainly focussed on issues concerning the measurement of continuity of care that had so far arisen in the research [13].

In 2008, the Service Delivery and Organisation commissioned a full and final synthesis of the studies from the present authors [14]. Our research had three main aims: to confirm or further refine the conceptual model of continuity of care developed and elaborated by Freeman and colleagues; to examine the findings to establish what influences continuity of care and what outcomes it leads to; and to examine how continuity of care was measured in the projects. In this paper we focus solely on the first aim, reporting the findings of a conceptual synthesis carried out using Critical Interpretive Synthesis, and considering the value of this novel approach. The results of the rest of the study are reported elsewhere [14–16].

## Methods

### Choice of critical interpretive synthesis

Various methods exist for synthesising purely qualitative research or studies based on mixed methods [17]. Meta-ethnography [18] is one of the most widely utilized and documented approaches [e.g., 19, 20]. In contrast, Critical Interpretive Synthesis is a relatively new approach to the synthesis of qualitative data [21]. While Critical Interpretive Synthesis draws on strategies from meta-ethnography, its developers see it as a unique approach with distinct advantages [21, 22].

As the name suggests, Critical Interpretive Synthesis is essentially an ‘interpretive’ mode of inquiry, the aim being to develop new concepts and theories through the process of review and synthesis. This differs from more ‘aggregative’ styles of review, which instead seek to compile and summarise the main findings of a body of evidence [23]. What distinguishes Critical Interpretive Synthesis from meta-ethnography and other approaches is its emphasis on theory-generation, its rejection of a ‘stage’ approach to review work, and its promotion of a more flexible, iterative, dynamic, critical and reflexive approach to synthesis [21, 22]. A particular advantage of this approach is that it enables researchers to engage critically with the assumptions underlying research which help shape and inform the results of studies in a given field. For these reasons, Critical Interpretive Synthesis seemed to offer the most potential for the present conceptual synthesis.

### Inclusion criteria

Final reports of the nine successfully completed projects carried out as part of the Programme were included in the conceptual synthesis. The final reports were obtained from the National Coordinating Centre for the Service Delivery and Organisation programme (NCCSDO), which had independently carried out peer-reviews of each of the reports prior to their publication. The initial scoping study and the interim review were also included, to examine the nature and development of the conceptual model of continuity of care elaborated through the course of the Programme.

We also read publications [24, 25] from a review of a contemporaneous parallel programme of work carried out for three Canadian health services organisations, with which the Service Delivery and Organisation Programme had links, in order to consider if and how this work had informed the ways in which continuity of care was conceptualised within the English Programme.

### Preparatory mapping of concepts

In preparation for the formal review, we each read all the final reports from the projects. One of the authors (JH) who took the lead on the conceptual review, identified places where authors had formally defined continuity of care. The definitions were drawn from the scoping study, which distinguishes six dimensions of continuity [2]; the severe mental illness project in which a revised version of this definition was reported, incorporating eight dimensions [11]; and from reports of the Canadian research in which a simpler tripartite definition was adopted [24, 25]. The nature of these definitions is examined in the results below.

While reading through all the final reports, JH began the process of identifying the various terms that were used to conceptualise continuity of care and these were discussed with the co-authors. This process revealed that a wide range of terms were used, including some derived from the scoping report, some from the revised version of the Freeman model, some from the Canadian review and other sources, and some were conceived by the authors themselves. The results of this preliminary mapping of the range and derivation of concepts itself suggested that the varied nature and use of the term continuity of care and associated ideas warranted further systematic exploration.

### Developing a critical interpretive synthesis

Unlike meta-ethnography, Critical Interpretive Synthesis has a relatively loosely defined set of processes for synthesising evidence. Essentially the approach requires a ‘lines of argument’ synthesis which results in a ‘synthesising argument’. Such an argument:

‘integrates evidence from across the studies in the review into a coherent theoretical framework comprising a network of constructs and the relationships between them’ and is ‘generated through a detailed analysis of the evidence included in a review, analogous to the analysis undertaken in primary qualitative research’ [21, p. 5 downloaded version].

Although it aims to demonstrate how its findings were generated using these processes, Critical Interpretive Synthesis does not claim or aspire to be reproducible by others. Rather, it acknowledges the ‘authorial voice’ of reviewers and recognises that:

‘alternative accounts of the same evidence might be possible using different authorial voices’ [22, p.39].

While using formal methods of data extraction is not integral to Critical Interpretive Synthesis, especially when working with larger bodies of literature [21], we made use of the ‘Framework’ approach, developed by Ritchie and Spencer [26] for analysing qualitative data, to facilitate the synthesis. Briefly, the Framework approach involves a process of familiarisation with data, developing a the matic framework and using this to index the data, and then abstracting and summarising the data in charts. Following this approach, we constructed a set of six charts based around the following themes: interpretation and use of the overall Freeman model of continuity of care; interpretation and use of the concept of ‘experienced continuity’ and related multi-axial concepts; the conceptualisation of different perspectives on continuity of care; the nature of ‘experienced continuity’ and its relationship to other concepts (such as patient satisfaction); the conceptual issues encountered or raised by the authors; and conceptual innovations. Relevant data were extracted and summarised in these charts by JH. Once completed, the charts were then checked and additional notes were added by the co-authors from their independent review of the reports. The final charts were then used to develop the Critical Interpretive Synthesis as follows:

In the first stage of developing the Critical Interpretive Synthesis, we examined how, if at all, the authors of each report drew on and developed the concepts originally outlined in the scoping report [2]. Where the authors used alternative concepts, we examined how these were derived and why they were preferred. The main purpose of this stage of analysis was to track the original conceptual underpinnings and progressive understanding, for each separate project. This included a focus on any issues that arose for projects in using the Freeman model in the course of their work.

In the second stage, we focussed more on the final conceptual positions adopted by the projects, examining similarities and differences in their stances, and triangulating the results with the original Freeman model of continuity of care [2]. The two main objectives here were to assess the extent to which the projects had eventually endorsed, modified or questioned the conceptual framework outlined in the scoping report, and to consider the nature of any modifications or revisions proposed by the researchers and how these related to an extended understanding of the concept of continuity of care.

In the final stage of analysis, we developed a more overarching interpretation of the findings that emerged from our review of the reports. This involved identifying and drawing together emerging themes from analysis of the conceptualisation of continuity of care within and across the nine studies, and triangulated with the Programme’s scoping report and interim review, as well as the reports from the Canadian Programme. In so doing, we hoped to come to a conclusion about the advances that had been made in the conceptualisation of continuity of care through the English Programme and, in turn, to add to this through our own synthesis. Further description of the methods used is provided elsewhere [14].

## Results

Here we describe the nature and use of the conceptual framework that underpins the Freeman model of continuity of care, followed by the more interpretive findings of our synthesis. For brevity, the individual studies are referred to by their patient group or topic (see Table 1).

### Conceptual framework underpinning the Freeman model

At the heart of the Freeman model of continuity of care are three propositions, each of which is outlined below.

#### Patients’ and carers’ experiences of continuity of care are what counts

In the scoping study, continuity of care is defined as:

‘The experience of a co-ordinated and smooth progression of care from the patient’s point of view’ [2, p.7].

While the term ‘carer’ does not appear in this definition, carers are included elsewhere in the report of the scoping study. Thus, in the Freeman model, patients’ and carers’ experiences of continuity are what count: their views must be examined in order to establish how they define and value it, and whether or not it was achieved from their perspective. From this proposition arises the notion of ‘experienced continuity’, which is a fundamental concept of the Freeman model and which permeates the Programme’s work.

#### Continuity of care is multi-dimensional

The second foundational claim of the Freeman model is that ‘experienced continuity’ is a complex, multi-dimensional concept. The achievement of good continuity from patients’ and carers’ perspectives largely depends on services doing well on the dimensions that are important to them. Freeman and colleagues [2, p.7] initially identified six dimensions of continuity in the scoping review—‘information’, ‘cross-boundary’, ‘team’, ‘flexible’, ‘longitudinal’, and ‘relational or personal’. These were later amended to eight in the severe mental illness project—‘relational’, ‘personal’, ‘therapeutic’, ‘longitudinal’, ‘flexible’, ‘information’, ‘cross-boundary’ and ‘team’ [11, p.31]. The Canadian review [25, p. 1220] proposed a simpler tripartite framework—‘informational’, ‘relational’, and ‘management’—which Freeman and colleagues [13] subsequently adopted as a general framework, while retaining the more detailed categories to distinguish different sub-types of continuity.

#### Processes and outcomes of continuity of care are both important

A third intrinsic, but less developed, claim underpinning the Freeman model is the need to consider patients’ and carers’ perspectives on both the processes and outcomes of continuity of care. This includes examining the consequences of continuity (or lack of it) on their health outcomes, as well as their satisfaction with the process of care.

### Use of Freeman’s conceptual framework

#### Perspectives on ‘experienced continuity’

We found the projects focussed mainly on patients’ perspectives on continuity of care. Carers’ views were also examined in the studies on primary care [3], mental health [4], diabetes [5] and cancer [8]. However, the ways in which carers were included here varied in two important respects.

First, there was no common definition of a carer used across the studies. Thus the ‘carers’ interviewed included some who were actively engaged in various aspects of care work and others who were less involved or not currently ‘caring’ as such. In the main cancer study, the authors acknowledged this by referring to this group as ‘close persons’ because of their varying caring relationship with the patients over time, although they retained the term ‘carers’ in the title of the report [8, p. 19]. And in the mental health study [4] it was acknowledged that fewer than two-thirds of the people interviewed as ‘carers’ regarded themselves as such.

Secondly, the capacity in which carers were interviewed varied. In the mental health [4] and diabetes [5] studies, carers were interviewed about their own experiences of continuity of care, in recognition that their views might differ from those of patients. The former study even developed a measure of carers’ ‘experienced continuity’. However, in the cancer study [8], carers’ proxy views on patients’ experiences of continuity were sought, as well as how patients’ experiences had affected them as carers. Their proxy views were compared with those of patients themselves to see if the assessments matched and provided a valid rating of patients’ ‘experienced continuity’. In the primary care study [3], carers’ perspectives were obtained and used to show how patients’ views and preferences were influenced and shaped by family experiences.

While professionals are not a central part of the Freeman model, their views were sought in all of the primary studies except one (primary care) [3]. Often the aim was to explore how professionals’ views converged with and diverged from those of patients and, to a lesser extent, carers. However, the diabetes study [5] went further and developed a measure of professionals’ ‘experienced continuity’. By prioritising the professional standpoint in this way, this study, along with the transition review [10], breaks somewhat with the emphasis on patients’ and carers’ perspectives in the Freeman model.

#### Dimensions of ‘experienced continuity’

Nearly all the projects attempted to investigate the extent to which the various dimensions of continuity of care elaborated by Freeman and colleagues (or the simpler version in the Canadian review) ‘mapped onto’ or corresponded with the views and experiences of the subjects of the studies. The projects on mental health [4], diabetes [5], stroke [7], cancer [8], NHS human resources management [12], and severe mental illness [11], found that there was some correspondence and concluded that the Freeman model was a useful framework, which they were able to add to and refine, using their own results.

For example, in the mental health study [4] and severe mental illness review [11], patients highlighted the importance of continuity of information exchange between patients and professionals. Previously, the dimension ‘informational continuity’ had been conceived as concerned with transfer of information and records between services and professionals only. Two new dimensions of continuity of care were also identified in the mental health study, namely ‘avoidance of services’ and ‘peer support’ [4, p. 267, p. 269]. And in the cancer study, the dimensions of ‘coping’ and ‘connections with family’ were added [8, p. 91].

However, in the stroke study [7] the researchers commented that it was sometimes difficult to map the concepts of continuity of care as articulated in the Freeman model. In the transition review [10], the researchers started with the dimensions in mind but ended up adopting another framework for organising their work and presenting the results. And in the report of the organisational and professional boundaries study [6], the authors made no reference to the Freeman model in one of their case conditions (stroke) but drew on a version of the framework from the Canadian review in the analysis of the other case (learning disabilities).

In their interim review of the Programme, Freeman and colleagues reflected that there was perhaps some ‘misunderstanding’ of the original model, resulting in a preoccupation with the dimensions rather than a focus on patients’ and carers’ own conceptualisations of continuity. They add that this preoccupation may have resulted in an expansion and further fragmentation of the multi-dimensional concept rather than improved knowledge of patients’ and carers’ own understanding of the meaning and importance of continuity of care. Hence they claim that they no longer think (if they ever did) that the outcomes of the various dimensions of continuity of care can be ‘packaged’ together into an overall measure or concept of ‘experienced continuity’ [13, p.47–49].

We also noted that none of the studies identified or suggested using patient- and carer-defined concepts in place of those from the model. Interestingly, in all the primary research studies (except mental health [4]), the researchers elected to avoid using the term ‘continuity of care’ directly in their discussions with patients and carers. It appears that the results of these discussions were simply mapped against the existing Freeman model and its associated dimensions to assess if the views expressed were in accord with the model or not, rather than being used to generate a new patient- and carer-defined conceptual framework or a re-working of the existing model.

#### ‘Experienced continuity’ as both process and outcome, and its relationship to other constructs

We observed a lack of clarity and consensus in the studies about whether continuity of care is a process and/or an outcome of care, and how outcomes can be measured from different perspectives. The projects on stroke [7], diabetes [5], cancer [8], primary care [3] and organisational and professional boundaries [6], also highlighted the related issue of whether or not the concept of ‘experienced continuity’ can be distinguished from constructs such as ‘patient satisfaction’, ‘patient-centeredness’ and ‘quality of care’ and, hence, if it can be measured as a distinct process and/or outcome. Furthermore, in the diabetes study [5], the authors argue that continuity of care is something to be valued in itself and not just for what difference it might make to clinical effectiveness.

### Critical interpretive synthesis: towards a paradigm shift?

Here we further expose the paradigm or world view underpinning the theories and methodology of the Freeman model. We show how the foundational claims of the original model represent a departure from the ways in which continuity of care was previously conceptualised, in terms of the new sets of perspectives that are prioritised. We also suggest that findings from the Programme may well signal a new emergent paradigm in this area.

#### The professional paradigm

With its emphasis on the centrality of patients’ and carers’ experiences of continuity of care, the Freeman model claims to differ from and improve upon previous conceptualisations of continuity of care which were instead almost entirely founded on professionals’ views. In what we shall refer to as the ‘professional paradigm’, continuity of care was regarded as a process that, with proper organisation and co-ordination of services and systems, could be delivered ‘to’ patients. Likewise, it was assumed that professionals have the most insight into the causes and factors that promote and hinder continuity of care; that discontinuity is a mark of failure in the system; that patients want to see the same professionals over time; and that patients and carers have relatively little influence over whether continuity of care is achieved or not.

#### The perspectivist paradigm

By contrast, in the Freeman model and in the studies that utilised its conceptual framework, it is accepted that ultimately, patients’ and carers’ experiences of continuity of care are what count, and that only they can define what it means to them and assess whether or not it was achieved. Patients, carers and professionals tend to be seen as each having their own separate, or proxy, perspectives on continuity of care, with patients the most privileged group.

In prioritising the patient’s point of view, and in recognising the discrete perspectives of patients’, carers’ and professionals’, the Freeman model represents a departure from the previous world view and a shift towards what we have termed a ‘perspectivist’ paradigm. In the new way of thinking, it is acknowledged that individual patients and carers may have personal and sometimes conflicting preferences and priorities, depending on their values. Professionals’ perspectives may also be sought and valued for what they reveal about their correspondence with patients’ and carers’ views, as well as providing an insight into organisational areas that are out with patients’ direct experience. Finally, it is assumed that patients and carers do not routinely use the same language of continuity of care as that of professionals.

By examining the perspectives of patients and carers, studies in the Programme were able to go beyond and show the limitations of the previous professional paradigm. As Freeman and colleagues speculated in the scoping report [2], for some patients, on some occasions, discontinuity was not found to be a negative experience. Thus, in the cancer follow-on study, some patients thought a break from service provision represented a return to ‘normality’ for a period [9, p. 61]. In the primary care study, some patients wanted to see different professionals in certain circumstances, such as when seeking help for embarrassing problems (in order to preserve their anonymity), or when they were dissatisfied with the opinion they had received from a previous doctor. The authors of this study further point out that ‘obligatory longitudinal continuity’ could impair the quality of care where professionals do not perform adequately [3, p. 17]. The review on transition [10] also stressed that change per se may not necessarily be a bad thing. Indeed, it suggested that change should be encouraged and facilitated in certain circumstances, for example, to help promote young peoples’ development and transition to adulthood.

#### The partnership paradigm

In the course of exploring patients’, carers’ and professionals’ perspectives, several studies reported findings that, we suggest, may well signal a new emerging paradigm that focuses less on the discrete perspectives of different parties (though these are still important) and more on the partnerships between patients, carers and professionals through which continuity of care is achieved as desired (or not). This confirms and extends the findings of the interim review [13], which also found strong support for the concept of patients as partners in their care.

In the partnership paradigm, the emphasis is placed on the connections and relationships between patients, their families and professionals, and the extent to which patients and informal carers are engaged as partners in care with professionals. Here it is assumed that continuity of care is co-constructed through the interaction between patients, members of their informal care networks and professionals. The achievement of good continuity depends on the strength of these connections and relationships. These themes were explicit in the findings of the studies on primary care [3], mental health [4], stroke [7], cancer [8], and transition [10].

The ways in which patients and informal carers are conceptualised likewise shifts from their being relatively passive recipients of care in the professional paradigm, and having individual preferences and priorities in the perspectivist paradigm, through to having agency, choice and control over defining and achieving continuity of care in the partnership paradigm. Here professionals do not so much deliver continuity of care ‘to’ patients as work ‘with’ them and their families to assess needs and preferences and to facilitate contact and continuity (and possibly change) of provision as appropriate. The more engaged patients and members of their informal care network are, the better for their continuity of care. It is recognised that, for some groups, professionals may have to be more proactive in identifying and working with people who are poorly connected and less engaged, and hence at risk of meeting barriers in accessing services and sustaining contact and continuity, with potentially negative consequences for their care and health outcomes.

A related point to note is that in the partnership paradigm the views and experiences of professionals are valued alongside those of patients and carers, as they are an important part of the partnership. This was emphasised in the studies on transition [10] and on NHS human resources management [12]. In addition, while the authors of the study on organisational and professional boundaries strongly asserted that only patients’ and carers’ views on continuity of care are meaningful, not professionals’ [6], elsewhere in their report they imply that continuity is a co-product of the relationship between patients, carers and the care system.

Finally, by exploring different perspectives on continuity of care across several conditions and services, the studies provided new insights into the lived experiences of patients and their carers, and their roles in negotiating and achieving continuity in different circumstances. For example, findings from the organisational and professional boundaries [6] and stroke [7] studies captured the complex nature and context of the journeys experienced by patients, carers and family members, and how these differed from, say, idealised ‘care pathways’. In so doing, some of the studies signal a more contingent and dynamic conceptualisation of continuity of care which more closely reflects the reality of the lived experience of patients and their families over time. Indeed, the stroke study referred to a more ‘dynamic’ model of continuity of care [7, p. 31–32], based on work by Donaldson [27], as a possible alternative to the conceptual model outlined in the scoping report but did not elaborate on this in their subsequent analysis.

## Discussion

We chose Critical Interpretive Synthesis because it seemed to offer the most potential for the conceptual part of the synthesis of the outputs of the Service Delivery and Organisation’s Continuity of Care Research Programme. In the end we felt that Critical Interpretive Synthesis was a good choice for this task. It enabled us to work at two levels: first, to review the nature of the conceptual framework and how it was used in the studies and, second, to analyse how the overall Programme had advanced understanding of the concept of continuity of care. The result of this synthesis was to indicate an emerging paradigm shift in this area, from one which prioritises the perspectives of patients and carers and sees them as distinct from professionals, to one which sees continuity as a product of the interaction between patients, carers and professionals, all of whom have an active part to play in its achievement.

While the concept of patients as ‘co-producers’ of care is not new (for example, in 1988 Dr. Julian Tudor Hart [28] championed a new social alliance model of working between doctors and patients as an alternative to the extant passive doctor–patient relationship), nonetheless this concept was not included in, nor a hallmark of, the original Freeman model of continuity of care, which itself was based on a scoping report of the extant literature on the subject [2]. Thus it may be that, by independently reiterating this theme and by extending it to include carers, the studies in the Continuity of Care Programme have made use of and developed the theme of co-production to the point where, if systematically taken up, it could form the basis of a new partnership paradigm.

A limitation of this work was that we did not interview key stakeholders, including members of the study teams, in the Programme (as Freeman did in his interim review [13]). Such interviews might have enhanced our understanding of the background to the Continuity of Care Programme, the formative influence of the scoping report and the Canadian work, and the working relationships and knowledge shared between the researchers who participated in the Programme. We did invite response to our draft report from all the study teams, but only one team took the opportunity to reply at length with further explanations, which were consistent with our analysis. We also did not review the wider literature on continuity of care ourselves because this had been done earlier in the Programme, as part of the scoping study that the Service Delivery and Organisation commissioned [2], and the funder’s brief was to synthesise the outputs of the Programme itself.

## Conclusions

Using Critical Interpretive Synthesis, we have shown how the studies in the Service Delivery and Organisation’s Continuity of Care Research Programme variously utilised a conceptual framework developed by Freeman and colleagues [2, 11]. In our higher-level synthesis, we have identified what we believe are signs of a shift towards an emergent ‘partnership’ paradigm which may provide the context for future research on this topic.

One of the strengths of the partnership paradigm is that there is a clearer focus on continuity of care as a complex and dynamic process of co-production involving patients, carers/families, and professionals. This has implications for the ways in which future studies might choose to investigate the achievement of continuity of care. For example, use of patient (and carer) reported outcome measures (PROMS) [29], as well as qualitative studies of their perspectives on the processes or means by which continuity was achieved or not, and the influence of ‘assets’ or social capital [30] that patients and carers/families may have (or not have) to engage with services, are all consistent with this way of thinking about continuity of care. So too is consideration of professionals’ part in the achievement of continuity. For example, doctors may also prefer to see the same patients to achieve better outcomes, as well as consideration of the policy and organisational issues that shape practice and people’s experiences of continuity of care.

The challenge for future studies operating within the partnership paradigm is to develop and utilise methods that enable exploration of not only what continuity of care means to patients, carers and professionals, and whether or not this is achieved, but *how* it is achieved by these groups and *why* it is achieved for some people and not others, and the social and organisational factors that promote or impede its achievement in particular contexts.

Finally, this critical interpretive synthesis, and the wider study of which it forms a part, focussed on a discrete programme of research work. In the overall study, we used different methods of synthesis to provide a comprehensive analysis of the conceptual, substantive and methodological findings of the projects from the Service Delivery and Organisation’s Continuity of Care Programme. This strategy may well be of interest to future research programmes, which as well as starting with a scoping review could consider ending with a formal synthesis of the outputs of the programme. As the present paper and our other reports on the work demonstrate [14–16], such a synthesis can provide valuable additional knowledge from the programme as a whole and help to take stock of the current state of understanding and identify future research priorities and strategies.

## Figures and Tables

**Table 1.** SDO Continuity of Care Research Programme Projects

Lead researcher/s, year of final report	Patient group/topic	Type of study
Baker et al. 2001 [3]	Primary care	Primary
Burns and Catty 2007 [4]	Mental health (2 linked studies, 1 report)	Primary
Gulliford et al. 2006 [5]	Diabetes (Type 2)	Primary
Hardy et al. 2005 [6]	Organisational and professional boundaries	Primary
Hill et al. 2008 [7]	Stroke	Primary
King et al. 2006 [8] and 2008 [9]	Cancer (2 linked studies, 2 reports)	Primary
Forbes et al. 2001 [10]	Transition from children’s to adult care for young people with chronic illness or disability	Review
Freeman et al. 2002 [11]	Severe mental illness	Review
Humphrey et al. 2002 [12]	NHS human resources management	Review

## Reviewers

**Line Melby**, PhD, Researcher, Institute of Health and Society, University of Oslo, Oslo, Norway

**Sir Denis Pereira Gray**, OBE, HonDSc FRCP FRCGP FMedSci, Emeritus Professor, University of Exeter; Honorary Professor, Peninsula College of Medicine and Dentistry, Alford House, 9 Marlborough Road, Exeter EX2 4TJ, UK

**Martin Roland**, Professor of Health Services Research, Institute of Public Health, Forvie Site, Robinson Way, Cambridge CB2 0SR, UK

**Rolf Wynn**, Professor, University of Tromsø, Norway
